# Disgust sensitivity and behavioural inhibitory systems in binge eating disorder: associations with eating pathology

**DOI:** 10.1007/s40519-023-01544-5

**Published:** 2023-02-20

**Authors:** Sarah L. Brassard, Michele Laliberte, James MacKillop, Iris M. Balodis

**Affiliations:** 1 Neuroscience Graduate Program, McMaster University, Hamilton, ON Canada; 2 Peter Boris Centre for Addictions Research, St. Joseph’s Healthcare Hamilton, 100 West 5th Street, Hamilton, ON L8P 3P2 Canada; 3 Eating Disorders Program, St. Joseph’s Healthcare Hamilton, Hamilton, ON Canada; 4 Michael G. DeGroote Centre for Medicinal Cannabis Research, Hamilton, ON Canada; 5 Department of Psychiatry and Behavioural Neurosciences, McMaster University, 1280 Main Street West, Hamilton, ON L8S 4L8 Canada

**Keywords:** Binge eating disorder, Behavioural inhibition, Disgust sensitivity, Eating pathology, Restraint, Go/No-Go

## Abstract

Disgust sensitivity refers to how unpleasant a disgusting experience is to an individual and is involved in the development of many psychiatric conditions. Given its link with food ingestion, there is an interest in understanding how an individual’s susceptibility to disgust relates to dietary habits. One possible mechanism giving rise to this association is through the effects negative emotions have on high-order cognitive processes, but few studies take this model into account. The aim of this study was to characterize general disgust sensitivity in a clinical binge eating disorder (BED) population, and explore whether disgust sensitivity relates to inhibitory control and eating pathology. Following a case-controlled study design, our results show that: (1) disgust sensitivity and its subscales do not differ between BED and healthy controls, (2) higher disgust sensitivity in BED relates to greater behavioural inhibition, (3) inhibitory control reaction times relate to aspects of eating pathology, and (4) inhibitory control does not mediate relationships between disgust sensitivity and BMI among participants with BED. Understanding the role of disgust sensitivity in BED may allow us to understand how negative emotion systems maintain dysregulated eating behaviours with the potential to inform emotion-regulation treatment approaches. *Level of evidence*: Level III: Evidence obtained from well-designed cohort or case–control analytic studies.

## Introduction

### Disgust sensitivity and inhibitory control

Disgust is a universally experienced primary emotion that serves to guide cognitive and behavioural functioning away from risk of infection and contamination by influencing eating cessation/food avoidance [1–3]. However, fairly little research has examined this construct to date. Behavioural components of disgust include modified facial expressions and actions that include stopping and dropping objects of disgust, shuddering or uttering “Ew!” [1, 2]. Its physiological components include nausea, gagging, lowered blood pressure and increased galvanic skin responses, which together can stop consummatory behaviours [4, 5]. Encompassing sensory and cognitive processes, disgust can influence eating cessation and avoidance of certain foods as well as other eating behaviours (i.e., anticipating how a certain food will taste, or the consequence of eating a certain food) [1], even without physical contact. For example, nausea, the most characteristic physiological manifestation of disgust, can influence food/eating avoidance without physical consumption [1]. Given this intimate link with food ingestion, there is an interest in understanding how an individual’s susceptibility to disgust relates to dietary habits.

One subfactor of disgust is disgust sensitivity—referring to how unpleasant a disgusting experience is to an individual [6]. With the capacity to rapidly link cognitive and affective processes, disgust sensitivity may also relate to the development and maintenance of psychiatric disorders [1]. To date, most research on disgust sensitivity has been in anxiety-related disorders [7–18], with additional investigations in avoidant behaviour [19], obsessive–compulsive behaviours [8, 20, 21] and social anxiety disorder [22, 23]. Very little research has investigated disgust sensitivity in eating disorders (EDs), and in binge eating disorder (BED) in particular [24]—one of the most commonly diagnosed EDs [25].

Given the universal nature of disgust sensitivity, this construct may be understood as a component of larger negative cognitive processes [1]. There is evidence that disgust sensitivity may be linked to both healthy and unhealthy eating [26]. Several disgust sensitivity theories suggest that this construct can serve as a protective mechanism preventing the consumption of risky foods with a potential high pathogen load (i.e., a rejection response surrounding eating) [3]. However, in the context of EDs, this construct may serve as an affective process associated with more restrictive eating behaviour (i.e., working in opposition to the hunger drive) [3, 26] driven by psychosocial factors influencing a person’s perspective of food and the body. Consistent with this second notion, disgust sensitivity relates to certain dysregulated eating behaviours, and eating pathology more generally [28–30]. Disgust sensitivity could move individuals away from a feared outcome different from infection—rather a fear of weight gain and/or feelings of fullness. While these hypotheses (protective anti-pathogen mechanism vs psychosocial affective process) are not mutually exclusive, they nevertheless represent different lenses for examining a possible (dys)functional role of disgust in eating behaviours—either through a general oversensitivity or acting as a defensive mechanism related to eating and weight domains.

### Disgust sensitivity in eating disorders and links with eating pathology

Rather than a generic process, disgust sensitivity across EDs may pertain primarily to areas concerned with food and the body [31, 32]. Indeed, relationships seem to exist between anorexia nervosa (AN) and bulimia nervosa (BN) and disgust related to animal foodstuff, and body-related stimuli. More specifically, in healthy adults and patients with AN, heightened disgust sensitivity associates with greater Drive for Thinness, Bulimia, Body Dissatisfaction, Ineffectiveness, Eating Disorders Inventory (EDI) total scores, and Eating Attitudes Test (EAT) total scores [29, 31]. Related to this notion (but looking at more psychological variables, rather than diagnoses), there is some evidence for relationships between restraint/drive for thinness and disgust sensitivity (especially to food). For instance, in clinical ED populations (including AN-restricting subtype, AN-binge/purge subtype, BN, eating disorder not otherwise specified and obese binge eaters) symptoms of AN measured via the Drive for Thinness subscale of the EDI also positively correlate with disgust sensitivity to Food and Magical Contagion [31]. Furthermore, higher Bulimia subscale scores of the EDI are associated with increased disgust sensitivity to Animals, Death, Body Envelope Violation and Magical Contagion, and marginally with overall Disgust Sensitivity [31]. In another clinical ED sample (diagnoses not specified), higher Drive for Thinness correlates with increased Core Disgust, Animal Reminder, Contamination-Based Disgust, Disgust Sensitivity, and higher Body Dissatisfaction correlates with increased Core Disgust, Contamination-Based Disgust and Disgust Sensitivity [33]. Collectively, these results suggest that disgust sensitivity to food/body-related stimuli appear related to measures of disordered eating—both to BN (measuring fear of loss of control over eating and compensatory efforts) and Drive for Thinness.

There are some mixed findings linking disgust constructs with eating behaviours and clinical measures. In nonclinical populations, a higher body mass index (BMI) is linked with decreased Core Disgust and Contamination-Based Disgust levels; whereas higher reported disgust levels are associated with increased restrained eating behaviours [34]. In further support of this association, heightened Core Disgust sensitivity relates to measures of self-disgust, motivating restrained eating behaviours with high BMI in non-obese individuals [26]. However, one early study did not find associations between food-related disgust and the avoidance of high calorie/highly palatable foods in a nonclinical sample of women [35]. Nevertheless, participants with AN or BN diagnoses show significantly higher levels of disgust sensitivity on Foodstuff of Animal Origin, Human Body Products, and Gastro-enteric Products subscale of the Disgust Questionnaire compared to a matched, nonclinical control sample [29].

For BED, there is currently no evidence for differences between clinical and non-clinical BED groups in general or food/body-related disgust sensitivity [24]. This is supported by neuroimaging studies demonstrated significant activation of the amygdala, insula and lateral orbitofrontal cortex (OFC) when viewing disgusting stimuli in all participant groups (BED, BN, overweight control subject and normal-weight control subjects)—but no significant group differences [36], suggesting similar activations during generic disgust processing across groups rather than binge-specific differences.

### Effects of negative emotions on executive functioning

Multiple theories on the development and maintenance of eating disorders suggest that negative emotions trigger engagement in emotional eating and binge eating episodes in both non-clinical and clinical populations [37, 38] by acting on executive functioning abilities. Current evidence suggests that the onset of  BN, characterized by cycles of binging and purging behaviours, co-occurs with periods of negative affect, suggesting associations between emotions and control over eating [39]. Furthermore, negative urgency—the tendency to act impulsively when feeling negative emotions—when combined with expectations that eating will alleviate negative affect, strongly characterises both BN [40, 41] and BED [42]. Based on a negative valence systems model of binge-type eating disorder risk [43], risk factors for binging behaviour (acting independently or in combination) include altered corticolimbic functioning, neuroendocrine dysregulation, and self-reported negative affect. Therefore, negative emotions like disgust may disrupt high-order cognitive processes like inhibitory control—a key feature in binge eating disorders [44–46]. For instance, emotions and inhibitory control share a ‘two-way connection’ between emotion processing and inhibitory control through shared brain networks (including the insula and inferior frontal gyrus) [47, 48], which allows emotions to disrupt inhibitory control and vice versa. The insula, which is heavily implicated in interoceptive awareness, emotional processing and response inhibition, may have the ability to ‘hijack’ self-control areas of the brain and affect inhibitory control via its projections that extend to various parts of the prefrontal cortex (housing numerous regions involved in inhibition) [49–54]. More specifically, the anterior insula acts as a “relay center” by receiving sensory information (from disgust-inducing stimuli), and subsequently modulates activity in response inhibition networks which consist of the left and right inferior and middle frontal gyri, right superior frontal gyrus, anterior cingulate cortex, anterior insula, subthalamic nucleus, pre-SMA, and dorsal aspects of the striatum [48, 49]. Therefore, disgust sensitivity may have the ability to indirectly influence eating behaviours via its effects on self-control processes; primarily by activating the anterior insula. Indeed, the effects of disgust sensitivity on BMI are mediated through reductions in food-specific inhibitory control [55].

Despite the effects negative emotions have on executive functioning, the relationship between inhibitory control and eating disorders is not straightforward. Response inhibition is generally assessed using validated stopping paradigms such as the Stop Signal Task and Go/No-Go tasks which require participants to withhold behavioural responses when a visual or auditory cue is presented. Impairments in inhibitory control across eating disorders differ between general and ‘disorder-salient’ stimuli (i.e., food/eating, body/shape). For generic stimuli, a meta-analysis of 5 Go/No-Go studies had non-significant effect sizes (Hedge’s *g* = − 0.39) across bulimic-type ED groups [56]. For food-specific inhibitory control stimuli, only one study showed significant inhibitory control deficits to food/eating stimuli (Hedges’ *g* =  − 0.68, *p* = 0.042) but not to shape/weight stimuli (*p* = 0.699) in BN patients [56, 57]. Greater deficits in inhibitory control to food/eating-related stimuli may therefore suggest that inhibitory control specifically to consummatory actions, may underlie recurrent binge eating episode [56]. Response inhibition findings in binge-type eating disorders are also mixed. For food-related inhibitory control, several studies show inhibitory control deficits in BED relative to normal weight and obese controls [57–59] with two others showing similar Go/No-Go performance to obese controls [60, 61]. One study found inhibitory control deficits to both generic (non-food related) and food-specific Go/No-Go tasks in participants with BED [62].

Dual-process models [63] of eating proposes that eating behaviour can be understood as the outcome of two different but complementary systems: self-regulatory processes such as inhibitory control (top-down processes) and automatic processes such as automatic appraisal of appetitive stimulus acquired through affective properties (bottom-up processes). Studies have recently begun to take dual-process model into account, based on the premise that automatic affective processes moderate the relationship between inhibitory control and eating behaviour. For example, Liu and colleagues found negative associations between disgust sensitivity and BMI, suggesting that undergraduate students with higher BMIs have lower sensitivity to disgust. Although contradictory to the dual-process premise, the associations between disgust sensitivity and BMI appear to be fully mediated by inhibitory control and not the other way around [55]. Nevertheless, Spinelli and colleagues found no associations between disgust sensitivity, restrained eating and BMI in individuals with obesity. However, higher levels of disgust sensitivity were associated with higher BMI in their sample of non-obese individuals, and this association appeared to be fully mediated by restrained eating behaviours [26]. Both studies were conducted in community samples, therefore it is not clear whether these findings generalize to clinical ED populations. Furthermore, investigations into how response inhibition and disgust sensitivity relate to eating pathology and loss of control eating remain to be examined.

### Current study

Considering that BED is the most common ED diagnosed, with lifetime prevalence ranging between 0.2 and 4.7%, and twelve-month prevalence estimates averaging 0.8% nationally [63], there is nevertheless a dearth of empirical studies examining disgust sensitivity in this population. The first aim of this study was to characterize disgust sensitivity in a clinical sample of patients with BED. Based on the findings that individuals with dysregulated eating behaviours exhibit similar levels of disgust to matched controls [28, 31, 36, 65], we hypothesized that individuals with BED would report similar levels of disgust sensitivity compared to healthy controls. If disgust sensitivity reflects more of an affective process associated with psychosocial aspects of eating behaviour or body image, then similar to previous AN and BN literature, we hypothesized that greater eating pathology (i.e., Restraint) would be associated with heightened (rather than lower) disgust sensitivity in our BED sample. In this way, aspects of disgust sensitivity would relate to attitudes around eating and body image domains, rather than BMI.

Given the links between disgust and inhibition, we also sought to explore whether disgust sensitivity might relate to inhibitory control in a BED population. Based on findings that disgust sensitivity is negatively correlated with inhibitory control in healthy controls [55] we hypothesized a similar relationship might exist in a BED sample. Next, we sought to extend associations between disgust sensitivity, behavioural inhibition and BMI to a clinical BED sample. Based on the finding that disgust sensitivity inversely related to BMI [34, 55], we also hypothesized that higher BMI would be significantly negatively associated with disgust sensitivity, and that behavioural inhibition would mediate the relationship.

## Methods

### Participants

Participants were 70 individuals (94.3% identifying as female gender) currently living in or around Hamilton Ontario, aged 20–64 (*M* = 41.35, SD = 13.01). Participants primarily identified as European (72.9%), with 4.3% identifying as South Asian (India, Sri Lanka, Pakistan, Nepal, Bangladesh), 5.7% as Native North American, 2.9% as Arab, 1.4% as Persian, and 12.9% as other. To take part in the study, participants were required to be at least 18 years of age. Healthy controls (*n* = 35) were required to have no current medical or psychiatric conditions, and no active or history of substance or alcohol use disorders. Participants with BED (*n* = 35) were recruited from the Eating Disorder Program at the St. Joseph’s Healthcare Hamilton West 5^th^ campus in Hamilton, Ontario, Canada. Healthy control participants were recruited from community advertisements, and matched to BED participants on sex and gender. Nine participants were removed from the dataset due to missing Go/No-Go data. A total sample size of 61 participants was used for our analyses (HC *N* = 32; BED *N* = 29).

### Procedure

The current study consists of data from part of a larger ongoing multi-session study examining reward and stress effects on decision-making and motivation. After obtaining informed consent, participants completed a battery of questionnaires including the MINI Psychiatric Assessment, the Disgust Scale-Revised, the Eating Disorders Examination Questionnaire, and the Beck Anxiety Inventory (descriptions below). Study sessions ended by completing the Go/No-Task. Study procedures were approved by the Hamilton Integrated Research Ethics Board (HiREB, Project 1600).

### Measures

#### Mini-International neuropsychiatric interview (MINI) [66, 67]

The Mini-International Neuropsychiatric Interview is a widely used structured clinical interview to assess major psychiatric disorders in the *Diagnostic and Statistics Manual* 3rd edition revised (DSM-III-TR), 4th edition (DSM-IV), 5th edition (DSM-V), and the *International Classification of Disease* 10th Revision (ICD-10). The MINI demonstrates excellent concordance with other structured clinical interviews including the Structured Clinical Interview for Diagnostic and Statistical Manual—patient version diagnoses (SCID-P) [68], and the Composite International Diagnostic Interview (CIDI) [64]. The MINI also demonstrates excellent interrater and retest reliability with Cohen’s kappa values ranging from 0.52 to 1.0 [66].

#### Disgust scale-revised (DS-R) [69]

The Disgust Scale-Revised is a 27-item self-report inventory modified from the initial 32-item Disgust Scale (DS) [27] designed to measure individual differences in disgust sensitivity. The DS-R assesses sensitivity to three disgust-eliciting domains including Core Disgust, Animal Reminder Disgust, and Contamination-Based Disgust. The DS-R demonstrates good internal validity (*α* = 0.87) with average inter-item correlations of 0.23. Internal consistency between the DS-R subscales is acceptable; with Core Disgust (*α* = 0.80, inter-item correlation = 0.23), Animal Reminder Disgust (*α* = 0.82, inter-item correlation = 0.34), and Contamination-Based Disgust (*α* = 0.71, inter-item correlation = 0.31). The DS-R also demonstrates good convergent and discriminant validity, with results suggesting that the three DS-R subscales are correlated, but not redundant [70]. Within the current sample, the DS-R demonstrated good overall internal consistency (*α* = 0.79), in addition to moderate internal consistencies in its Core Disgust (*α* = 0.51), Animal Reminder Disgust (*α* = 0.67), and Contamination-Based Disgust (*α* = 0.61) subscales.

#### Eating disorder examination questionnaire (EDE-Q) [71]

The Eating Disorder Examination Questionnaire is a self-report version of the Eating Disorder Examination (EDE), and was designed to measure the attitudinal and behavioural features of patients with EDs. Primarily used for diagnostic purposes, subjects are required to report how frequently they engage in a certain pathological eating behaviour based on a 7-point scale from 0 (no days) to 6 (every day) within the past 28 days. The EDE-Q examines eating pathology across four subscales include Restraint, Eating Concern, Shape Concern and Weight Concern. A Global score is also calculated as a mean across all four subscales. The EDE-Q has good concurrent validity, and acceptable criterion validity, with correlations between EDE-Q and EDE subscales ranging between 0.68 and 0.78 [72]. Test–retest reliability ranged from 0.66 to 0.94 for scores on the four subscales, and from 0.51 to 0.92 for items addressing the frequency of behavioural engagement. The EDE-Q demonstrates acceptable internal consistency, with alpha’s ranging from 0.70 to 0.93 [73]. Internal consistency for the EDE-Q in the current sample was high, with an alpha of 0.92.

#### Beck anxiety inventory (BAI) [74]

The Beck Anxiety Inventory (BAI) is a widely used 21-item self-report measure of anxiety severity in adolescents and adults. Items are scored in a Likert scale fashion ranging from 0 (not at all) to 3 (severely, I could barely stand it). The BAI shows high internal consistency (*α* = 0.92) and test–retest reliability over one week [*r*(81) = 0.75] [74]. The BAI also shows good concurrent and discriminant validity. In the current sample, the BAI demonstrated a high internal consistency with an alpha of 0.95.

#### Body mass index (BMI)

BMI was calculated based on participants self-reported height (in metres) and weight (in kg) using the standard formula (BMI = kg/m^2^ where kg is a participant’s weight in kilograms, and m^2^ is the participants height in metres squared). Participants BMIs ranged from underweight to obese (*M*[SD] = 33.98 [11.42]; range = 16.2–69.1), with 2.8% of participants having a BMI in the underweight range (BMI < 18.5), 18.3% in the normal weight range (BMI = 18.5–24.99), 19.7% of participants in the overweight range (BMI = 25.0–29.99), and 56.3% in the obese range (BMI > 30.0) (missing data *N* = 2). Height was recorded in centimeters using a stadiometer located in a dietitian’s office, and weight was recorded using a yearly calibrated digital bariatric scale.

### Go/No-Go task

The Go/No-Go task is cognitive task aimed at determining the ability of an individual to inhibit a prepotent response. The experimental paradigm requires participants to make responses when they see a ‘Go’ signal, and withhold a response when they see a ‘No-Go’ signal. The main dependent variable in the Go/No-Go task are commission errors (making a ‘Go’ response on ‘No-Go’ trials); fewer errors signify better response inhibition [75].

### Statistical analysis

Data were screened for outliers and normality. A one-way ANOVA was conducted with the EDE-Q and its subscales (DV: EDE-Q Total Score, Restraint subscale, Eating Concern subscale, Shape Concern subscale and Weight Concern subscale; IV: participant group) to test group differences in eating pathology. To test for group differences in disgust sensitivity and behavioural inhibition, we conducted two separate one-way ANCOVAs, while controlling for anxiety. The first ANCOVA tested group differences on the Disgust Sensitivity Total Scores; the second ANCOVA tested for group differences on Commission Errors, the primary dependent variable of the Go/No-Go task. Next, we conducted two exploratory MANOVAs with each of the DS-R subscales as well as Go/No-Go parameters between our samples (MANOVA 1 DVs: Core Disgust score, Animal Reminder score, Contamination-Based score; MANOVA 2 DVs: Omission Errors, Trial Reaction Times and ‘Go’ Reaction Time; IV: participant group). Given the high prevalence of anxiety-related symptoms in BED [76], the current study controlled for anxiety symptomatology with the BAI. Although the primary aim of our study was to test the relationship between eating pathology and associations with behavioural and emotional measures of inhibition, we explored each subscale that may be driving effects in instances where significant differences emerged. To assess whether eating pathology is associated with behavioural and/or emotional inhibition in our BED sample, Pearson Correlations were conducted between the EDE-Q subscales and total score, the DS-R subscales and total scores and each parameter of the Go/No-Go task. To adjust for anxiety, Partial Correlations were run using the same measures as our Pearson Correlations, but controlling for BAI scores. Lastly, to test whether BMI was associated with cognitive and/or behavioural inhibition, correlational analyses were conducted between BMI, DS-R and parameters from the Go/No-Go task first within, then between groups. A mediation model was also tested using Model 4 in Hayes’ PROCESS macro for SPSS [77]. The significance of the indirect effect of disgust sensitivity on BMI through inhibitory control was evaluated using the 95% confidence intervals calculated from 5000 bootstrapped samples. Four models tested the following: (1) the indirect effect of Disgust Sensitivity total score on BMI via number of Commission Errors, (2) the indirect effect of Core Disgust on BMI via number of Commission Errors, (3) the indirect effect of Animal Reminder on BMI via number of Commission Errors, and (4) the indirect effect of Contamination-Based Disgust on BMI via number of Commission Errors. Analyses were conducted using IBM SPSS 26.0 (IBM Corp. Armonk, NY).

## Results

### Demographics and descriptive statistics

Table 1 presents the demographic characteristics of our sample, followed by mean scores on the EDE-Q between our participant groups. As expected, the BED group had a significantly higher BMI compared to our control group and differed in ED pathology, with significantly higher EDE-Q total scores compared to controls. Our sample with BED scored significantly higher on Eating Concern, Shape Concern and Weight Concern compared to our healthy control group, but not on the Restraint subscale.

**Table 1:** Demographics

	BED (*N* = 29)	HC (*N* = 32)
Demographics		
Gender	1 Males 28 Females	2 Males 30 Females
Ethnicity	82.8% European 3.4% South Asian 3.4% Native North American 10.3% Other	62.5% European 9.4% Native North American 6.3% Arab 6.3% South Asian 3.1% Persian 12.5% Other
Highest level of education	72.4% university degree	81.3% university degree
Employment status	69% currently employed	75% currently employed
	Mean (SD)	Mean (SD)
Age	44.82 (12.09)	39.29 (13.94)
Height (cm) (SD)	166.55 (9.03)	168.61 (8.58)
Weight (kg) (SD)**	115.01 (28.91)	75.64 (21.17)
BMI**	41.43 (9.14)	26.54 (7.17)
BAI**	21.03 (14.04)	6.65 (7.58)
EDE-Q		
EDE-Q total score**	3.02 (1.38)	1.28 (1.19)
Restraint	1.39 (1.41)	0.91 (1.32)
Eating concern**	2.59 (1.65)	0.53 (1.13)
Shape concern**	4.18 (1.60)	1.96 (1.57)
Weight concern**	3.92 (1.62)	1.71 (1.47)

Data presented as M (SD) unless otherwise noted

*BMI* body mass index, *BAI* beck anxiety inventory, *EDE-Q* eating disorders examination questionnaire

*p < 0.05, **p < 0.01 two-tailed significance

### Disgust sensitivity

**Fig. 1:**
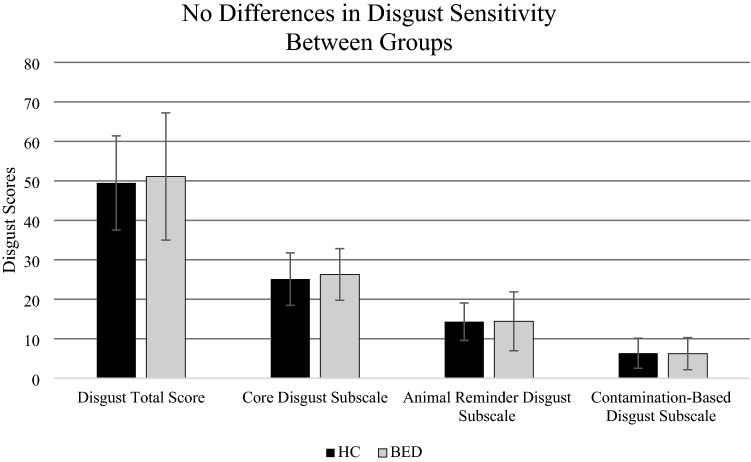
Mean scores on the revised Disgust Sensitivity Questionnaire by participant group. Mean scores are shown for Disgust Sensitivity Total Score, Core Disgust Subscale, Animal Reminder Subscale and Contamination-Based Subscales of the DS-R for binge eating disorder (BED, *n* = 29) and healthy control (HC, *n* = 32) participants

No significant group difference emerged following our one-way ANCOVA, controlling for anxiety, between healthy controls (*M* = 49.84, SD = 11.26) and participants with BED (*M* = 52, SD = 15.84) on mean Disgust Sensitivity Total Scores [*f*(58) = 0.225, *p* > 0.05] (Fig. 1). An additional exploratory MANCOVA revealed no significant differences on the Core Disgust subscale [*f*(58) = 0.595, *p* > 0.05], on the Animal Reminder Disgust subscale [*f*(58) = 0.001, *p* > 0.05], nor the Contamination-Based Disgust subscale [*f*(58) = 0.047, *p* > 0.05].

### Behavioural inhibition

No significant group difference on commission errors emerged following our one-way ANCOVA between healthy controls (*M* = 3.78, SD = 2.51) and participants with BED (*M* = 4.03, SD = 2.64) on the Go/No-Go [*f*(58) = 0.673, *p* > 0.05] after controlling for anxiety. An additional exploratory MANCOVA of secondary Go/No-Go measures revealed no significant differences on the mean omission errors [*f*(58) = 0.421, *p* > 0.05], on Trial reaction time [*f*(58) = 1.117, *p* > 0.05], nor ‘Go’ mean reaction times [*f*(58) = 0.480, *p* > 0.05].

### Associations between disgust sensitivity and behavioural inhibition

**Fig. 2:**
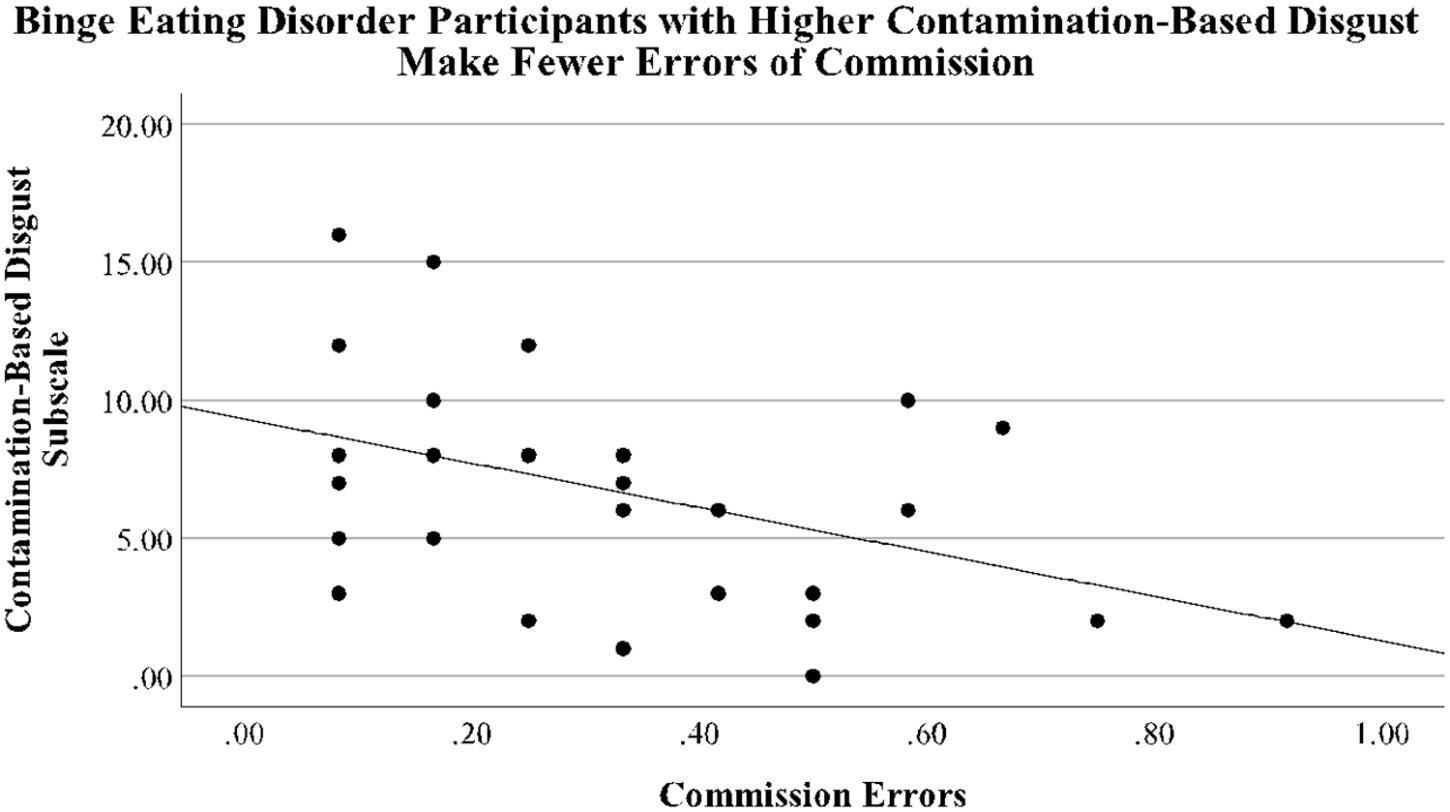
Pearson Correlation shows a significant negative association between Contamination-Based Disgust and Commission Error in participants with Binge Eating Disorder

Within our BED participants, a negative association between Disgust total score and Commission Errors was trending significance in our BED sample (*r* = − 0.365, *p* = 0.052), suggesting that BED with higher disgust sensitivity are better at making accurate responses on the Go/No-Go task. Further examinations into the different Disgust subscales demonstrated that Contamination-Based Disgust Subscale scores significantly negatively correlated with Commission Errors on the Go/No-Go Task (*r* = − 0.435, *p* = 0.018), suggesting that BED with higher Contamination-Based disgust sensitivity have better response inhibition (Fig. 2). No significant correlations emerged in our sample of healthy controls, nor after collapsing groups together. Results remained the same after removing male subjects from our analyses.

### Associations between disgust sensitivity, behavioural inhibition and eating pathology

Pearson correlation tests showed no significant associations between EDE-Q Total Scores and measures of disgust sensitivity, or behavioural inhibition within our BED participants (Table 2). Exploring the EDE-Q subscales showed a positive correlation between the Restraint subscale and the ‘Go’ Mean Reaction Time of the Go/No-Go task (*r* = 0. 386, *p* < 0.05; Table 2), suggesting participants with higher restraint took longer to make behavioural responses on ‘Go’ trials. After controlling for anxiety, this correlation remained significant. Restraint and ‘Trial’ Reaction Time (*r* = 0.38, *p* < 0.05) also became significantly positively associated suggesting that participants with higher restraint took longer to make behavioural responses on every trial (‘Go’ and ‘No-Go’ trial; Table 3). Shape Concern and Core Disgust (*r* = 0.394, *p* < 0.05; Table 3) additionally became significantly positively correlated after controlling for anxiety, suggesting that BED participants with greater shape concerns experience greater sensitivity to disgust based on the offensiveness and the threat of disease (e.g. arising from rotten foods and waste products). In our sample of healthy controls, EDE-Q total score significantly correlated with Disgust Total Score (*r* = − 0.35, *p* < 0.05), further subscale exploration showed that the Restraint subscale of the EDE-Q significantly correlated with Disgust Total Score (*r* = − 0.436, *p* < 0.05) and Contamination-Based Disgust (*r* = − 0.478, *p* < 0.05). Results remained the same after removing male subjects from our analyses.

**Table 2:** Exploratory correlations between measures of disgust sensitivity, behavioural inhibition and eating pathology in a BED sample

	EDE-Q total score	Restraint	Eating concerns	Shape concern	Weight concern
Disgust total score	0.274	0.207	0.19	0.309	0.257
Core disgust	0.277	0.174	0.12	0.354	0.323
Animal reminder	0.174	0.169	0.114	0.212	0.121
Contamination-based	0.331	0.251	0.349	0.27	0.291
Commission errors	− 0.243	− 0.315	− 0.195	− 0.118	− 0.241
Omission errors	0.232	0.08	0.245	0.263	0.214
‘Trial’ reaction time	0.042	0.364	− 0.027	− 0.108	− 0.037
‘Go’ mean reaction time	0.085	0.386*	0.013	− 0.06	− 0.001

*EDE-Q* eating disorders examination questionnaire

*p < 0.05

**Table 3:** Partial correlations between measures of cognitive inhibition, behavioural inhibition and eating pathology after controlling for anxiety in BED sample

	EDE-Q total score	Restraint	Eating concerns	Shape concern	Weight concern
Disgust total score	0.29	0.219	0.198	0.319	0.262
Core disgust	0.327	0.219	0.156	0.394*	0.354
Animal reminder	0.15	0.144	0.085	0.194	0.1
Contamination-based	0.347	0.261	0.366	0.274	0.294
Commission errors	− 0.213	− 0.292	− 0.161	− 0.084	− 0.216
Omission errors	0.134	− 0.046	0.15	0.189	0.145
‘Trial’ reaction time	0.028	0.38*	− 0.047	− 0.128	− 0.051
‘Go’ mean reaction time	0.049	0.38*	− 0.029	− 0.097	− 0.03

*EDE-Q* eating disorders examination questionnaire

*p < 0.05

### Associations between disgust sensitivity, behavioural inhibition and BMI

Within the HC group, Pearson correlation coefficients showed no significant associations between BMI, disgust sensitivity or its subscales, or Go/No-Go parameters. Similarly, no significant associations emerged between BMI, disgust sensitivity and Go/No-Go parameters in our participants with BED. Results from our mediation models show that inhibitory control does not mediate any relationships between disgust sensitivity and BMI (Table 4).

**Table 4:** Mediation models testing the hypothesis that inhibitory control mediates the association between disgust sensitivity and BMI in BED

	Estimate	SE	*t*	*p*	95% CIs
*Model 1: disgust total score/BMI*					
Disgust total score and commission error	− 0.005	0.003	− 1.92	> 0.05	–
Commission error and BMI	− 4.11	8.63	− 0.47	> 0.05	–
Direct effect: disgust total score and BMI	− 0.11	0.12	− 0.89	> 0.05-	− 0.36 to 0.14
Indirect effect: disgust total score and BMI via commission error	0.02	0.04	–		− 0.05 to 0.10
*Model 2: core disgust/BMI*					
Core disgust and commission error	− 0.01	0.006	− 1.75	> 0.05	–
Commission error and BMI	− 3.25	8.60	− 0.38	> 0.05	–
Direct effect: core disgust and BMI	− 0.197	0.29	− 0.67	> 0.05	− 0.79 to 0.40
Indirect effect: core disgust and BMI via commission error	0.04	0.07	–	–	− 0.11 to 0.18
*Model 3: animal reminder disgust/BMI*					
Animal reminder and commission error	− 0.008	0.006	− 1.33	> 0.05	–
Commission error and BMI	− 2.42	8.43	− 0.29	> 0.05	–
Direct effect: animal reminder and BMI	− 0.13	0.26	− 0.51	> 0.05	− 0.67 to 0.40
Indirect effect: animal reminder and BMI via commission error	0.019	0.06	–	–	− 0.10 to 0.15
*Model 4: contamination-based disgust/BMI*					
Contamination-based and commission error	− 0.02	0.01	− 2.39	0.02	–
Commission error and BMI	− 6.81	8.70	− 0.78	> 0.05	–
Direct effect: contamination-based and BMI	− 0.69	0.48	− 1.46	> 0.05	− 1.68 to 0.29
Indirect effect: contamination-based and BMI via commission error	0.16	0.17	–	–	− 0.17 to 0.52

## Discussion

The aim of this study was to characterize general disgust sensitivity in a clinical BED population. We found no group differences in self-reported disgust sensitivity levels between individuals with BED and healthy controls. Nevertheless, within the BED group, higher disgust sensitivity related to greater behavioural inhibition, driven by contamination-based disgust levels with commission errors. Further exploration of the behavioural data showed positive associations between Go/No-Go reaction times and Restraint levels on the EDE-Q. No associations were found between disgust sensitivity and eating pathology in our sample of BED participants. Lastly, a mediation model did not find that inhibitory control, as measured via commission errors on the Go/No-Go task, mediated any relationships between disgust sensitivity and BMI among participants with clinical BED.

Our results suggest that individuals with BED do not differ from healthy controls in overall levels of disgust sensitivity. Further analyses of disgust subscales revealed similar levels of on all three disgust domains (Core Disgust, Animal Reminder Disgust, and Contamination-Based Disgust) as our healthy participants. While previous relationships between disgust sensitivity and EDs have been examined (in AN, [28, 32]; in BN, [32, 68]; in restrictive eating and obesity [34]; and in ARFID [78]), our study is the first to investigate disgust sensitivity in a clinical sample of BED patients. This study also replicates and extends findings from Schienle and colleagues [36] who found similar disgust sensitivity levels to healthy individuals in a community sample of individuals with BED. Our results are, therefore, consistent with previous findings of disgust sensitivity in other EDs, in both clinical and nonclinical samples. Moreover, these findings support the idea that most EDs are not characterized by a disgust over- or under-sensitivity. If disgust sensitivity reflected affective process associated with psychosocial aspects of eating behaviour or body image, we would have expected to see greater eating pathology associated with heightened disgust sensitivity in our BED sample; our null results suggest that neither diagnosis nor severity of symptoms in BED patients (eating or body image) are related to disgust stimuli that are not specific to ED concerns. Future studies examining more food- and shape-specific disgust may shed light on particular aspects of disgust more directly related to ED pathology, and allow examination of possible links with dysregulated eating behaviours.

We also support our second hypothesis that disgust sensitivity is negatively correlated with general inhibitory control by demonstrating a negative trending association between Total Disgust, particularly driven by Contamination-Based Disgust Subscale scores and Commission Errors on the Go/No-Go Task in our sample of BED patients. These results extend previous reports in undergraduate students and non-obese individuals using food-related stimuli demonstrating an inverse relationship between disgust sensitivity and behavioural inhibition [26, 55] suggesting that general, non-food-related disgust sensitivity may have the ability to indirectly influence eating behaviours via its effects on self-control processes. Taking a closer look at associations between behavioural inhibition and eating pathology, our results suggest that BED participants with higher EDE-Q Restraint scores also took significantly longer to make ‘Go’ and ‘No-Go’ responses suggesting a possible high-order conscientious influence on self-control processes. Interestingly, no associations were observed between disgust sensitivity and measures of eating pathology among BED participants. Other studies assessing the effects of negative affect on cognitive functioning in binge eating disorders have also demonstrated that in individuals with binge-type eating disorders (namely, BED and BN) have greater impairments in decision-making abilities following negative affect compared to healthy controls [79, 80]. Disgust is unique in this study as it is a negative emotion that also incorporate elements of arousal considering its close ties with anxiety-related disorder, and our results may support theories of binging as a way to escape/avoid negative emotions. Nonetheless, understanding the interaction of emotional and cognitive processes is important to better understand the development and maintenance of binge eating disorder, and foster the development of improved clinical interventions. Future research examining disgust stimuli that is more relevant to BED (i.e., larger body image, more decadent and palatable foods) may shed light onto stronger relationships between disgust sensitivity, eating pathology, and impulse control.

In contrast to our hypothesis and previous research in non-BED groups, our results suggest that higher BMI is not associated with disgust sensitivity levels in BED. Despite Houben and Havermans [34] proposing lower disgust sensitivity encouraging overeating behaviours, the lack of association between high BMI and disgust sensitivity is consistent with the idea that disgust may be more closely linked to affective processes. Our findings nevertheless suggest a relationship between disgust sensitivity and eating attitudes; in particular a role for the severity of body image concerns in BED, a factor known to be associated with greater distress and poor treatment outcomes in BED [81]. Research in a nonclinical population showed that the desire to binge is greater when experiencing higher levels of body dissatisfaction/distress [82]. These findings highlight the cognitive-affective link with emotion regulation whereby different disgust-related eating features may have a unique maintenance function on eating attitudes, including the severity of shape and weight concerns. In contrast to previous studies [26, 55], we found no mediating role of behavioural inhibition when further examining associations between disgust sensitivity and BMI, suggesting differentials roles between cognitive and behavioural inhibition across EDs. Indeed, whereas Spinelli and colleagues [26] found no associations between disgust sensitivity, restrained eating and BMI in individuals with obesity, they did find associations between higher levels of disgust sensitivity and higher BMI in their sample of non-obese individuals, which appeared to be fully mediated by restrained eating behaviours [26, 55]. While BMI may be a marker for more dysregulated eating and/or less restriction, our sample is mostly obese and dysregulated in their eating, and as such we may be looking at a limited restricted range. Relationship between BMI, disgust and impulse control may only arise in a broader non-obese samples.

### Strengths and limitations

This is the first study to assess disgust sensitivity in a clinical sample of individuals with BED. We extended findings of disgust sensitivity patterns previously reported in AN and BN to BED, while controlling for baseline anxiety. Specifically, we replicated associations between disgust sensitivity (related to contamination-based disgust), and behavioural inhibition. The current study did not find a relationship between BMI and disgust sensitivity, nor did it find a mediating role of behavioural inhibition. Additionally, despite using different measures of disgust sensitivity and eating pathology (namely the revised Disgust Scale and the EDE-Q), we found similarities in our results with previous ED research, therefore extending the validity between measures to the reproducibility of consistent results. This study is also unique in examining general traits and processes (i.e., generalized disgust and inhibitory control), rather than food or disorder-specific stimuli.

Our findings should be considered in light of some limitations. First, although double the size of the first study examining disgust sensitivity in a community BED sample [36], our sample size is still relatively small and powered to only detect medium to large effect size relationships; nevertheless, we have replicated and extended previous findings from other ED studies to a clinical BED sample. Second, the individual DS-R subscales show lower internal consistency, which may be due to the sample size or type (i.e., clinical) or from different versions of the measure. Future revisions of these subscale seems warranted. Third, although sex differences in disgust sensitivity are evident [83], these could not be addressed as our sample had a higher representation of females compared to males (with only 4 males included altogether). This is important to highlight as cognitive-affect processes may also differ between sexes and could be examined with a larger and more diverse sample. Future research could assess disgust sensitivity in a community sample of males with dysregulated eating behaviours as a preliminary way to assess sex differences in a large sample, as males are often underrepresented in eating disorder treatment settings. Furthermore, our cross-sectional study makes it difficult to assess temporal interrelationships between disgust sensitivity and eating pathology. Although disgust sensitivity is often conceptualized as a trait, state-dependent testing will also be important for future research to assess affective processes, including craving, loss of control eating and body image triggers, linked to immediate states. Lastly, given the characteristics of our sample, matching healthy control participants to BED patients on BMI was not possible. As such, differences in body muscle/fat compositions exist between individuals within each group, which may relate to other between-group differences and our underlying constructs of interest. Our sample sex-ratio is also a limitation; our study was also conducted only with females, and differences may emerge with males in our sample or an all-male study.

### Future directions

Future research should consider replicating these findings in larger clinical samples and further differentiating between food and non-food items using a food-specific disgust measure, as disgust sources may have an effect on the development and maintenance of eating disorders. Additionally, future studies could assess the extent of food-related disgust in the context of different levels of body image distress/dissatisfaction, or after an experience of loss of control over eating. Future studies should also consider further differentiating between moral aspects of disgust. For example, examining self-disgust and behavioural disgust, as feelings of disgust towards the self is a DSM-5 characteristic required for a BED diagnosis [84], and appears elevated in individuals with binging behaviours (ex; BN) [85]. Furthermore, a study in an undergraduate population suggests that negative body image relates to higher self-disgust scores, heightened disgust propensity and disgust sensitivity, and that people who experience disgust more readily are more likely to experience disgust directed towards themselves [86]. Importantly, von Spreckelsen and colleagues [86] found that only disgust sensitivity (as opposed to self-disgust or disgust propensity) appeared independently related to a negative body image—further substantiating a link between these two constructs. However, associations stemming from this approach may be context dependent; if you’ve violated your eating rules versus general self-disgust. Additionally, examining food-related inhibitory control, for example through an affective Go/No-Go task, can also test whether stronger associations exist with disgust. Further replication and extension of these results with greater sample sizes in clinical disordered eating populations will be valuable, particularly as they relate to persistent body dissatisfaction and changes over treatment.

### Conclusion

This study is the first to preliminarily assess disgust sensitivity in a clinical BED sample and examine subsequent associations with behavioural inhibition. Findings from our study support relationships between disgust sensitivity and inhibitory control, and further suggest potential cognitive-affective linkages formed in maintaining BED pathology. We replicated associations between disgust sensitivity and eating pathology reported in other EDs—specifically on similarities in overall disgust sensitivity between BED patients and healthy controls. Examining disgust sensitivity in BED can shed light on how affective processing and inhibitory control systems might maintain dysregulated eating behaviours.

## What is already known on this subject?

Disgust is a universally experienced emotion that has strong ties with consummatory behaviour. Evidence suggests that relationships exist between anorexia nervosa and bulimia nervosa and disgust related to food and body-related stimuli, as well as between restraint/drive for thinness and disgust sensitivity (particularly to food stimuli). For binge eating disorder, there is currently no evidence for differences between clinical and non-clinical BED groups in general or food/body-related disgust sensitivity, nor a clear understanding of how self-regulatory processes and affective processes influence binge eating behaviours.

## What does this study add?

To the best of our knowledge, this is the first study to characterize general disgust sensitivity in a clinical binge eating disorder population. We add to the existing disgust in eating disorders literature by demonstrating that general disgust sensitivity does not differ between BED patients and healthy individuals, and that behavioural inhibition relates to disgust sensitivity but does not mediate relationships between disgust sensitivity and BMI in patients with BED.
